# Immune priming prior to pathogen exposure sheds light on the relationship between host, microbiome and pathogen in disease

**DOI:** 10.1098/rsos.220810

**Published:** 2023-02-01

**Authors:** Alyssa W. Kaganer, Robert J. Ossiboff, Nicole I. Keith, Krysten L. Schuler, Pierre Comizzoli, Matthew P. Hare, Robert C. Fleischer, Brian Gratwicke, Elizabeth M. Bunting

**Affiliations:** ^1^ Department of Natural Resources and the Environment, College of Agriculture and Life Sciences, Cornell University, Ithaca, NY, 14853, USA; ^2^ Smithsonian's National Zoo and Conservation Biology Institute, Washington, DC, 20008, USA; ^3^ Cornell Wildlife Health Laboratory, Animal Health Diagnostic Center, Cornell University, Ithaca, NY, 14853, USA; ^4^ Department of Population Medicine and Diagnostic Sciences, College of Veterinary Medicine, Cornell University, Ithaca, NY, 14853, USA; ^5^ Department of Comparative, Diagnostic, and Population Medicine, College of Veterinary Medicine, University of Florida, Gainesville, FL, 32611, USA; ^6^ Biology Department, Hamilton College, Clinton, NY, 13323, USA; ^7^ Smithsonian's National Zoo and Conservation Biology Institute, Front Royal, VA, 22630, USA

**Keywords:** emerging infectious disease, host–pathogen interaction, amphibian, transcriptome, microbiome, *Batrachochytrium dendrobatidis*

## Abstract

Dynamic interactions between host, pathogen and host-associated microbiome dictate infection outcomes. Pathogens including *Batrachochytrium dendrobatidis* (Bd) threaten global biodiversity, but conservation efforts are hindered by limited understanding of amphibian host, Bd and microbiome interactions. We conducted a vaccination and infection experiment using Eastern hellbender salamanders (*Cryptobranchus alleganiensis alleganiensis*) challenged with Bd to observe infection, skin microbial communities and gene expression of host skin, pathogen and microbiome throughout the experiment. Most animals survived high Bd loads regardless of their vaccination status and vaccination did not affect pathogen load, but host gene expression differed based on vaccination. Oral vaccination (exposure to killed Bd) stimulated immune gene upregulation while topically and sham-vaccinated animals did not significantly upregulate immune genes. In early infection, topically vaccinated animals upregulated immune genes but orally and sham-vaccinated animals downregulated immune genes. Bd increased pathogenicity-associated gene expression in late infection when Bd loads were highest. The microbiome was altered by Bd, but there was no correlation between anti-Bd microbe abundance or richness and pathogen burden. Our observations suggest that hellbenders initially generate a vigorous immune response to Bd, which is ineffective at controlling disease and is subsequently modulated. Interactions with antifungal skin microbiota did not influence disease progression.

## 1. Introduction

Emerging infectious diseases (EIDs) have broad-ranging impacts on the health of humans, domestic animals and wildlife [1,2]; they are driven by planetary changes including globalization and climate change [3,4]. Tools to mitigate EIDs are difficult to find because of complex, context-dependent interactions between a host, host-associated microbiome, pathogen and shared environment that affect the transmission and pathogenesis of disease [5,6].

Global amphibian populations are experiencing rapid declines and over 40% of known species are threatened with extinction [7–9]. One of the primary threats to amphibians are EIDs [10–12], and one fungal pathogen *Batrachochytrium dendrobatidis* (Bd) has decimated global amphibian populations, causing significant declines and extinctions [11,13–15]. Bd disrupts critical amphibian skin functions, leading to morbidity and mortality [16,17], but infection outcomes vary due to Bd strain, environmental factors, composition of skin microbial communities and host susceptibility [18–22].

Amphibian immunity includes non-specific innate immune responses that are shared with other vertebrates, including phagocytic cells and an antibody-dependent complement system [23]. Crucially, the unique structure of amphibian skin confers innate defences not found in other vertebrates and arguably most relevant to cutaneous pathogens like Bd. The skin mucus contains antibodies, proteolytic enzymes, phagocytic Langerhans cells and anti-microbial peptides manufactured by granular glands, which are released in response to stress [23,24]. The amphibian skin microbiome is the community of commensal microbes which are directly affected by these skin defences and by Bd infection, but many microbial taxa themselves secrete antifungal metabolites that inhibit Bd and may be regarded as part of the innate defence system [25,26]. Inhibition of Bd is functionally redundant within the skin microbiome of amphibians and higher proportions of anti-Bd bacterial taxa may be associated with decreased host susceptibility to Bd [27,28]; however, microbiomes are challenging to experimentally manipulate [22,26,29].

In addition to innate immunity, amphibians respond to Bd infection with acquired immune mechanisms which are triggered following pathogen recognition by antigen-presenting cells, leading to activation of Class I and II major histocompatibility complex (MHC) molecules and related antibody secretions [23,30]. Amphibian skin is active in adaptive immunity; B cells in the skin produce mucosal antibodies [31] and MHC antigens are upregulated in skin following pathogen exposure [32–34]. Expression of the MHC is a particularly critical component of amphibian response to Bd infection in many species and is linked to improved infection outcome in several species [32,33,35–37]. While effective activation of an acquired immune response has been linked to Bd resistance in some amphibian species [31,32,38–40], Bd appears capable of evading clearance by inhibiting immune functions [41].

Efforts to bolster the acquired immune response via immune priming have been applied to many amphibians with variable results, suggesting that the specific role of acquired immunity in disease resistance may vary by host species [31,39,42–45]. An early Bd vaccination experiment in resistant *Xenopus laevis* produced an antibody response to intraperitoneal injection of killed Bd [31]. However, subcutaneous and intraperitoneal injections of killed Bd and an adjuvant administered to susceptible *Rana muscosa* and *R. sierrae* failed to produce a detectable antibody response or reduce the probability of infection or death [42,46]. Subsequent studies have evaluated the effect of immune priming on host response to Bd infection in animals topically exposed to Bd who cleared infection and then were re-exposed [32,43,44]. These experiments have yielded inconsistent results among susceptible species; the outcome of Bd re-exposure in *Litoria booroolongensis* and *Atelopus zeteki* was not improved by immune priming [32,43], but *Osteopilus septentrionalis* had improved outcomes in Bd re-exposure with decreased Bd load and increased splenic lymphocyte abundance and proliferation [44]. It is not clear whether topical exposure to Bd can induce a protective mucosal antibody response; this remains an important research topic and a potential mitigation method [23].

Independent functional characterization of host, host-associated microbes or Bd during infection have contributed greatly to our understanding of the individual roles that each component plays during disease [31–33,35,38,46–51]. Functional genomic tools have been especially valuable to characterize and understand host responses [32,38,40,52], or pathogen gene expression at a single time point in infection [51]. As a generalist pathogen, Bd is known to alter gene expression rapidly both *in vitro* and *in vivo* to effectively parasitize diverse hosts [53–55]. This results in altered pathogen function when exposed to amphibian skin, which is probably both species-specific and context-dependent, including upregulation of virulence-associated protease, chitin binding and membrane transport genes, and downregulation of phosphorylation and protein kinase genes associated with generalized cellular metabolism [49,51].

In order to better understand the interaction between an amphibian host, pathogen and microbiome, we examined the relative roles of all three components simultaneously at several points during an infection cycle. We chose to specifically study host skin because it is an immune organ in amphibians and is accessible for longitudinal sampling, which is not possible for other immune organs such as spleen. Our host, the Eastern hellbender salamander (*Cryptobranchus alleganiensis alleganiensis*), was an appropriate choice for a longitudinal metatranscriptomic study of gene expression of the host, Bd pathogen and microbiome in the skin because these large-bodied salamanders have an extensive tail margin. This experiment focuses on the site of infection; the tail margin both represents a body region with the greatest density of chytridiomycosis lesions in urodeles [56] and practically permits the multiple skin biopsies required for longitudinal experimental design. Wild hellbenders are not considered to be highly susceptible to chytridiomycosis and can maintain subclinical Bd infections for long periods of time [57–63]. However, the Cornell Wildlife Health Laboratory confirmed clinical chytridiomycosis as a cause of mortality in hellbenders submitted from the New York State Department of Environmental Conservation (NYSDEC) captive rear-and-release programme in 2014 (E.M.B, personal communication), suggesting that Bd-naive captive hellbenders are susceptible to clinical chytridiomycosis. We hypothesized that acquired immunity is responsible for the differential disease outcomes between Bd-exposed wild hellbenders and Bd-naive captive hellbenders. To evaluate the role of acquired immunity in hellbender chytridiomycosis, we conducted an experimental vaccination and challenge trial.

## 2. Materials and methods

### 2.1. Vaccination and challenge

#### 2.1.1. Experimental procedures

**Figure 1.**
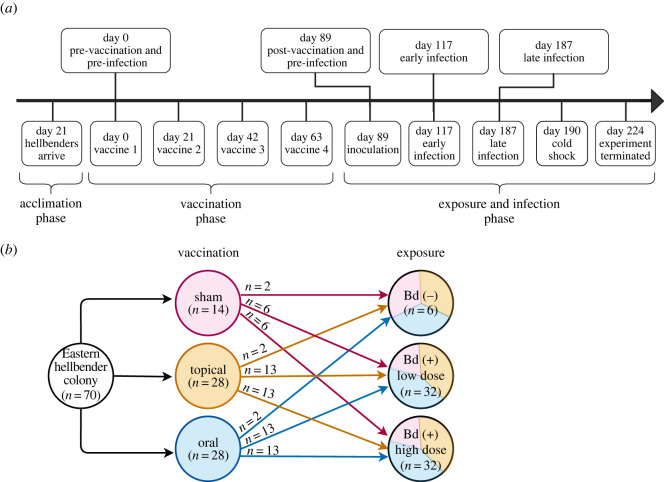
Experimental vaccination and Bd infection of Eastern hellbender salamanders. (*a*) Timeline of experimental procedures: animals were acclimatized for 21 days, received a series of four vaccinations over 89 days with killed Bd, then were inoculated with live Bd and monitored for 135 days. (*b*) Experimental design: animals were randomly assigned to vaccination groups, first vaccinated with either oral, topical or sham control doses of killed Bd, then randomly assigned to an exposure group and infected with either a high, low or sham control dose of Bd.

Eastern hellbender salamanders (*n* = 70) were sourced from the NYSDEC hellbender captive rear-and-release programme. All animals were reared for 6 years by the Buffalo Zoo from eggs collected from a stream within the Allegheny watershed. Hellbender mean mass was 324.8 g and ranged from 177 g to 538 g at the beginning of the experiment. Sex was not determined prior to inclusion in the study because sex cannot be reliably determined in this species with external examination. All animals were individually identified by passive integrated transponders and were confirmed Bd-naive prior to enrolment in the study. Hellbenders were transported to the Cornell University College of Veterinary Medicine and acclimatized for 21 days in eight living stream tanks with six–eight animals per tank (figure 1*a*). In order to test the different mechanisms through which wild hellbenders could be exposed to Bd naturally, either topically through contact with contaminated water, or orally through ingestion of contaminated prey, we individually vaccinated hellbenders with one of three randomly assigned treatments: (i) liquid nitrogen-killed Bd administered topically by pipetting onto flank skin (*n* = 28), (ii) liquid nitrogen-killed Bd administered orally by pipetting into the oral cavity (*n* = 28) or (iii) sham vaccination with deionized water (dH_2_O, *n* = 14; figure 1*b*). All vaccination inoculum consisted of a 0.5 ml solution of dH_2_O and 1 × 10^6^ killed Bd zoospores (strain JEL422, isolated from *Sachatamia albomaculata*). To ensure that topical vaccine inoculum was not immediately washed from the hellbender skin, animals were placed in individual bins (16 Quart Sterilite container), lightly rinsed with tank water and held for 2 h following the vaccination treatment before being placed back into the tank. To control for administration effects all animals received both a topical and an oral treatment, their assigned vaccination with killed Bd inoculum and an alternative sham inoculum. Vaccination protocols were repeated three times at 21-day intervals for a total of four treatments administered over 63 days (figure 1*a*).

To assess the effect of exposure dose on Bd infection progression in vaccinated hellbenders, we randomly assigned hellbenders to experimental challenge groups and each animal received either a high (*n* = 32, 0.5 ml dH_2_O and 1 × 10^6^ zoospores), low (*n* = 32, 0.5 ml dH_2_O and 1 × 10^3^ zoospores) or sham (*n* = 6, 0.5 ml dH_2_O) inoculation of live Bd (JEL422, figure 1*b*). All live Bd inoculations were administered topically to flank skin and left to incubate in individual containers for 2 h before being placed back into tanks. Survival and infection progress in all animals was monitored for 135 days post Bd infection (DPI). All infected animals and three sham-infected animals were euthanized on experimental day 224 (135 DPI) with an intracardiac injection of sodium pentobarbital at 100 mg kg^−1^ and necropsied at the conclusion of the experiment.

All animals experienced an accidental 6–9°C drop in water temperature from previously maintained temperatures of 18–21°C on experimental day 190 (101 DPI, figure 1*a*). This resulted from mechanical failure of both main and backup boilers in the aquatic animal facility. Following discovery of the cold shock, all tanks were returned to standard temperatures within 24 h. We selected all samples for functional genomics analysis prior to the cold-shock period to avoid having to interpret the role of this unintended experimental factor in genomics results.

In order to characterize the functional role of host, pathogen and the commensal microbiome in vaccination and infection, we sampled hellbenders throughout the experiment to test for Bd load, evaluate the metatranscriptome of host, pathogen and commensal microbiome, and characterize the commensal skin microbial communities. Animals were swabbed with two sterile polyester-tipped applicators following standard techniques [64,65]. The first swab was stored dry at −20°C for Bd load quantification, the second swab was stored in RNAlater (Invitrogen, Carlsbad, CA, USA) at −80°C for 16S rRNA microbiome analysis. Swab samples were collected prior to each experimental manipulation during the acclimatizion and vaccination phases, and twice per week during the exposure & infection phase (day 89 to day 224, figure 1*a*). We also took two 3 mm punch biopsies of skin from the tail margin: (i) fixed in 10% neutral buffered formalin for histopathologic examination to characterize evidence of chytridiomycosis, (ii) preserved in RNAlater at −80°C for gene expression analysis of host, pathogen and skin microbes. Biopsies were collected prior to each experimental manipulation during the acclimatizion and vaccination phases, and once every two weeks during the exposure & infection phase (day 89 to day 224, figure 1*a*). All animal handling and sample collection protocols were approved by the Cornell University Institutional Animal Care and Use Committee (Protocol ID: 2015–0045).

#### 2.1.2. Evaluation of hellbender disease status

Complete post-mortem examinations were performed on 67 hellbenders, including all Bd-inoculated animals (*n* = 64) and three negative control animals. Samples of major visceral organs and skin from five sites (tail, distal thoracic limb, distal pelvic limb, gular region and flank) were fixed in 10% neutral buffered formalin. Serial formalin-fixed tail-punch biopsies from 29 hellbenders, and post-mortem tissue samples from all hellbenders were processed routinely, stained with haematoxylin and eosin, and examined microscopically for evidence of chytridiomycosis. For punch biopsy histologic analysis, we prioritized samples from every Bd (−) animal (*n* = 6), and animals that died or were euthanized during the study (*n* = 2), then randomly selected low-dose Bd (+) animals that received sham, topical and oral vaccinations (*n* = 3, each), and selected high-dose Bd (+) animals that received sham (*n* = 6), topical (*n* = 3) and oral (*n* = 3) vaccinations (figure 1*b*).

### 2.2. Pathogen quantification

We extracted DNA from skin swabs collected for Bd load analysis using PrepMan Ultra (Applied Biosystems, Foster City, CA, USA) and diluted extracts 1 : 10 in MilliQ water (ThermoFisher, Waltham, MA, USA) [66]. The presence and severity of Bd infection was determined with a quantitative PCR (qPCR) assay targeting the fungal ITS1 sequence of the Bd genome [67] and quantified using a commercially synthesized (Integrated DNA Technologies, Inc, Coralville, IA, USA) ssDNA fragment containing primer and probe binding sites. All qPCR results are reported in units of ITS1 copy number to account for variability in ITS1 copy number among Bd strains [68] because ITS1 copies per genome have not been characterized for JEL422. We controlled for contamination at qPCR by including negative control reactions of dH_2_O template in every PCR run.

All statistics were performed in R v. 4.0.2. We tested for an effect of vaccination and infection dose on Bd presence and load with a linear mixed effects model using the R package lme4 [69]. Our model evaluated total Bd ITS1 copies over time for each animal, which was represented by area under the disease progression curve (AUDPC) [70]. We calculated AUDPC per animal with the trapezoidal rule by summing the area of the trapezoids generated between sequential measurements in a plot of Bd ITS1 copies over time. The final model included AUDPC of all Bd (+) hellbenders (*n* = 64) as response variable, with vaccination and infection dosage as fixed effects and tank as a random effect. We tested the significance of fixed effects in the linear mixed model with a Kenward–Roger's F test implemented with the lmerTest R package [71].

### 2.3. Host, pathogen and host-associated microbiome functional genomics

#### 2.3.1. General metatranscriptomics

We investigated the functional genomics of hellbender skin, Bd and the microbiome during vaccination and infection with an RNA sequencing (RNA-Seq) approach. For RNA-Seq extractions, we prioritized skin punch biopsies from every Bd (−) animal (*n* = 6) and every Bd (+) animal that received a sham vaccination (*n* = 6). We completed our sample library with randomly selected Bd (+) animals that received either an oral vaccination or topical vaccination (*n* = 3, each). Sequencing libraries were prepared for each selected animal at four time points: (i) pre-vaccination (experimental day 0), (ii) post-vaccination (experimental day 89), (iii) early infection (experimental day 117, 28 DPI), and (iv) late infection (experimental day 187, 98 DPI, figure 1*a*).

Total RNA was extracted from hellbender skin punch biopsies stored in RNAlater using a modified TRIzol extraction protocol (ThermoFisher). Each biopsy sample was macerated in TRIzol, and nucleic acids were phase separated with 1-bromo-3-chloropropane (Sigma-Aldrich, St Louis, MO, USA). Total RNA was precipitated from the aqueous phase with isopropanol and GenElute-LPA carrier (Sigma-Aldrich). Precipitated total RNA was washed with ethanol and suspended in RNA Storage Solution with Superase RNase inhibitor (Invitrogen). Total RNA was DNase treated with Turbo DNA-free (ThermoFisher), quantified with Qubit RNA HS assay (Invitrogen) and visualized with Bioanalyzer RNA Pico Assay (Agilent Technologies, Santa Clara, CA, USA) [72]. Total RNA extracts were rRNA depleted with a modified Ribo-Zero Gold Epidemiology (Illumina, San Diego, CA, USA) protocol by Clontech Laboratories (Mountain View, CA, USA) [73]. All rRNA-depleted samples were cleaned with RNAclean XP (Agencourt Bioscience, Beverly, MA, USA) and cDNA libraries were prepared with ScriptSeq v. 2 (Illumina) following the manufacturer's protocol. Sequencing libraries were quantified with Qubit DNA HS assay (Invitrogen) and visualized with Bioanalyzer High Sensitivity DNA Assay (Agilent Technologies). Libraries with greater than 5% of the sample composed of adapter dimer were size-selected with BluePippin (Sage Science, Beverly, MA, USA) and cleaned with Ampure XP (Beckman Coulter, Indianapolis, IN, USA). All libraries were qPCR quantified with KAPA Illumina Library Quantification Kit (Roche, Pleasanton, CA, USA) and combined in an equimolar pool. The pooled library was treated with Illumina Free Adapter Blocking Reagent (Illumina) and submitted to Novogene (Sacramento, CA, USA) for sequencing on a NovaSeq 6000 S4 flowcell (Illumina) with 150 bp paired-end reads.

All sequencing analysis was performed on the Smithsonian Institution High Performance Computing Cluster (Smithsonian Institution, https://doi.org/10.25572/SIHPC). Read quality was assessed with FastQC v. 0.11.8 upon receipt from the sequencing core and following each subsequent quality filtration step [74]. Raw reads were error corrected with Rcorrector [75]. Corrected reads were adapter and quality trimmed with Trimmomatic v. 0.39 [76]. We trimmed Illumina adapter sequences, trimmed bases at 3′ and 5′ read ends with quality below Q20, and scanned reads with a 3 bp sliding window, truncating reads if quality score fell below Q20. Finally, we filtered all reads less than 18 base pairs long after trimming. Trimmed reads were then filtered for rRNA sequences in two iterative steps; first with SortMeRNA v. 2.1b against the default rRNA database which includes 16S, 23S, 18S and 28S rRNA sequences [77,78]. The second filtration was performed by comparing the remaining reads against a custom database containing the genome of the PhiX control spike in (GenBank accession NC_001422), complete rRNA sequences (SILVA release 132) and tRNA sequences (GtRNAdb database) [79,80]. Filtration was performed using blastn implemented in BLAST+ v. 2.6.0 with a minimum alignment bit score of 54 [78,81,82]. Resulting reads were filtered for human and viral contaminants with Kraken2 by comparison against the default database with confidence parameter 1 [83]. All contaminating reads were removed with BBMap v. 38.67, (sourceforge.net/projects/bbmap/). The final cleaned mRNA database was separated into prokaryote, Bd and hellbender reads by first removing all prokaryote sequences with Kraken2 comparison with the default database with default settings. The remaining eukaryote mRNA reads were aligned to the Bd genome (JEL423, GenBank accession GCA_000149865.1) with permissive parameters using STAR v. 2.7.5c with a seed length of 18 nucleotides and requiring a minimum of 30% bases matching to reference [84]. Eukaryotic reads that successfully aligned to the Bd genome were considered Bd-derived reads. Eukaryotic reads that failed alignment to the Bd genome were considered hellbender-derived reads.

#### 2.3.2. Host functional genomics

The hellbender skin transcriptome was de novo assembled with Trinity v. 2.11.0 using default parameters [85]. Transcript abundance was estimated per sample with RNA-Seq by expectation maximization (RSEM) [86]. Large quantities of both transcripts and samples rendered standard count matrix generation infeasible; therefore, we adopted a bootstrapping approach to filter transcripts. We first randomly selected 60 samples and generated a normalized count matrix with RSEM implemented in Trinity. We then selected transcripts with expression support of at least one read per million mappable reads in at least one sample. We repeated randomly selecting subsets of 60 samples, generating count matrices and filtering by expression for a total of 10 bootstrap cycles. We sequentially compared lists of highly expressed transcripts after each bootstrap and retained all newly identified transcripts such that any transcript identified in any bootstrap was included in a final list of filtered transcripts. A count matrix was generated from the final list of filtered transcripts. To remove low-level expression noise, we filtered out transcripts with less than two reads per million mappable reads in at least two samples [87,88]. The remaining transcripts were aligned to the National Center for Biotechnology non-redundant protein (nr) database using the blastx application within the BLASTx program v. 2.4.0, retaining up to 20 hits with a minimum E-value of 1 × 10^6^ [81,82]. We functionally annotated the transcriptome with BLAST2GO command line v. 1.4.4 using default parameters and retained all transcripts with significant blastx alignments or gene ontology (GO) term mapping for expression analysis [89]. The raw count matrix of remaining transcripts was converted to counts per million to correct for differences in library size and filtered to remove background expression noise, retaining only genes with at least five reads mapping per million mappable reads in at least one sample. The filtered count matrix was normalized with the trimmed mean of m-values to account for variation in library composition, and tagwise dispersion was estimated. We used principal components analysis to evaluate clustering of hellbender skin functions throughout vaccination and Bd infection.

To identify changes in hellbender skin function in response to vaccination and Bd infection, we analysed differential gene expression (DGE) in edgeR with a quasi-likelihood negative binomial generalized log-linear model of the expression counts. We accounted for the two types of error (1—between animals and 2—between repeat measures of the same animal) in our multi-level experimental design by initializing our model by animal and treating all measurements from pre-vaccination samples as a shared baseline. All pairwise comparisons for DGE are listed in the electronic supplementary material, table S1. Genes with a log fold change (FC) > 1 and false discovery rate (FDR)-corrected *p* < 0.05 were considered significantly differentially expressed. We tested for functional enrichment in biological processes identified during DGE analysis with two-sided Fisher's exact tests for over- or under-represented processes with a FDR cut-off of 0.05 implemented in BLAST2GO [90]. Each Fisher's exact test was performed with a test set of differentially expressed genes (DEGs) and the reference set of all functionally annotated hellbender skin genes. We characterized the expression of immunity-associated genes in hellbender skin by retaining only genes annotated with the immune system process (GO:0002376) or any of its child GO terms among the DEGs. We used a heatmap of centred and scaled Log_2_ FC expression values using pheatmap [91] to visualize changes in immunome-wide expression from baseline pre-vaccination expression.

To clarify whether the expression of MHC genes was affected by vaccination strategy or Bd status, we queried the list of DEGs identified in each experimental group at each time point for genes with MHC annotation as identified by BLAST2GO. We modelled longitudinal expression for each MHC gene with vaccination strategy and Bd status as two nested whole plot factors and experimental time point as a subplot factor in f2.ld.f1 linear models implemented in nparLD [92]. We used ANOVA-type tests of the f2.ld.f1 models to identify effects of vaccination and infection on MHC expression.

#### 2.3.3. Pathogen functional genomics

We compared the function of Bd during early and late infected hellbenders by analysing expression of the Bd-derived reads identified during hellbender skin transcriptome preparation. The pool of Bd reads was aligned again to the Bd genome with more stringent parameters using STAR v. 2.7.5c with a seed length of 18 nucleotides and default filtering constraints [84]. Read counts per genomic feature were quantified with featureCounts implemented in Subread v. 2.0.1 with fractional counting of multi-mapped reads [93,94]. Raw read counts for each sample were aggregated and prepared for DGE analysis with edgeR following the methods described above [95,96]. We accounted for repeat measures of expression in early and late infection by initializing our model design matrix by animal and treating early infection expression values as baseline. We identified DEGs between early and late infection following the methods described above. All pairwise comparisons investigated for Bd DGE are listed in the electronic supplementary material, table S2. We used principal components analysis to evaluate clustering between Bd in early and late infection by vaccination strategy. Differential expression was visualized with a heatmap using normalized log-transformed *z*-scores of count data in pheatmap [91].

We identified enriched Bd functions between early and late infection with Fisher's exact tests as described above. The Bd genome was functionally annotated with blastx and BLAST2GO using the methods detailed previously. Functional enrichment was identified using a test set of DEGs and the reference set of all functionally annotated Bd genes.

Finally, we investigated Bd pathogenicity mechanisms that may have contributed to the observed increase in Bd load during late infection. We retained DEGs with putative pathogenesis function as identified by BLAST2GO and compared their expression between early and late infection with a paired *t*-test. To identify whether Bd pathogenicity was altered in vaccinated hosts, we compared the expression of pathogenicity genes between early and late infection within each vaccination group with a Kruskal–Wallis test.

#### 2.3.4. Host-associated microbiome functional genomics

We additionally evaluated the function of the hellbender skin microbiome by analysing expression of putative prokaryote-derived reads identified during hellbender transcriptome preparation. The prokaryote metatranscriptome was de novo assembled with Trinity v. 2.11.0 using default parameters [85]. Transcript abundance was estimated and a count matrix was generated with RSEM implemented in the Trinity pipeline [86,97]. We visualized gene expression patterns with principal component analysis in edgeR [96].

Microbiome function was then characterized within experimental groups by experimental time point. Transcripts were subdivided by vaccination and infection experimental group at pre-vaccination, post-vaccination, early infection and late infection; any transcript with an abundance count greater than 0 was included in the respective dataset. We quantified the overlap in observed biological process GO terms among datasets with overlapping Venn diagrams in VENNY v. 2.1 [98]. Redundancy in assigned GO terms was reduced by day with REVIGO; terms with a similarity score of less than or equal to 0.5 were retained [99].

### 2.4. Host-associated microbiome characterization

We evaluated the effects of vaccination and infection on hellbender skin microbial communities with a 16S rRNA sequencing approach. We selected every sham-infected animal (*n* = 6) and every animal infected with a high dose of Bd (*n* = 32) in the vaccination experiment for 16S rRNA analysis. We extracted DNA from hellbender skin swabs with a DNeasy Blood and Tissue Kit following the manufacturer's protocol with pre-treatment for gram-positive bacteria (Qiagen, Valencia, CA, USA). A sterile swab was included in each batch of extractions to act as a negative control. Extracted DNA from all samples and controls was prepared for 16S rRNA amplicon sequencing following previously described methods [100,101]. Amplicons from all samples and negative controls were quantified with Qubit dsDNA HS assay kit (Invitrogen, Carlsbad, CA, USA) and pooled in equimolar quantities by sample. The resulting library was size-selected with QIAquick Gel Extraction Kit and purified with QIAquick PCR Purification Kit (Qiagen, Valencia, CA, USA). The purified library was visualized with a Bioanalyzer High Sensitivity DNA chip (Agilent Technologies, Santa Clara, CA, USA) and quantified with the KAPA Illumina Library Quantification Kit (Roche, Pleasanton, CA, USA). The library was spiked with 15% PhiX and sequenced on an Illumina MiSeq with v. 2 chemistry and 2 × 250 bp paired-end reads [101].

Raw sequencing reads were adapter trimmed, joined, demultiplexed and assigned to samples with MacQIIME version 1.9.1 [102]. Joined reads were quality filtered by removing all reads with one or more expected errors using USEARCH v. 10.0.240 [103,104]. High-quality sequences were clustered to operational taxonomic units (OTUs) at 97% similarity using UPARSE implemented in USEARCH [105]. Clustered OTUs were filtered to remove rare variants comprising greater than 0.001% of counts [106]. Taxonomy was assigned to a representative sequence from each OTU with the RDP classifier at 80% confidence threshold against the Greengenes database (2013) [107–109]. Representative sequences were aligned with PyNAST, and a phylogenetic tree was built using FastTree2 [110,111]. All OTUs which failed to align or which were identified as contaminants in control samples were removed from downstream analysis.

To evaluate the effect of vaccination, we compared microbiome alpha and beta diversity between vaccination groups at two time points, pre-vaccination and post-vaccination using 16S rRNA sequencing. Alpha diversity was measured with Faith's phylogenetic diversity (PD) index and beta diversity was measured with the Bray–Curtis distance [112]. Raw sequence count data were normalized prior to calculating Bray–Curtis distance to account for biases associated with uneven sequencing depth between samples [113].

We compared Faith's PD index among experimental time points with paired Wilcoxon signed-rank exact tests. To evaluate vaccination or Bd infection effects on Faith's PD index metrics or variance, we tested for differences among experimental groups during each time point with (i) Kruskal–Wallis tests and (ii) Fligner–Killeen tests for homogeneity of variance. Finally, we identified experimental factors impacting alpha diversity across all experimental time points with a linear mixed effects model. Our model included Faith's PD index as the response variable, vaccination treatment and infection dose as both independent and interacting fixed effects, and animal as a random factor. Experimental factors impacting beta diversity at each experimental time point were identified with PERMANOVA of the Bray–Curtis distance using the vegan package in R [114].

We characterized the presence and abundance of putative anti-Bd bacterial taxa by filtering OTUs and retaining only known culturable anti-Bd bacterial taxa [18]. To evaluate the relationship between anti-Bd bacterial taxa and Bd load in hellbenders, we tested for correlation between richness and abundance of anti-Bd OTUs and Log_10_Bd ITS1 copy number using a Spearman's rank correlation.

## 3. Results

### 3.1. Experimental Bd challenge

**Figure 2.**
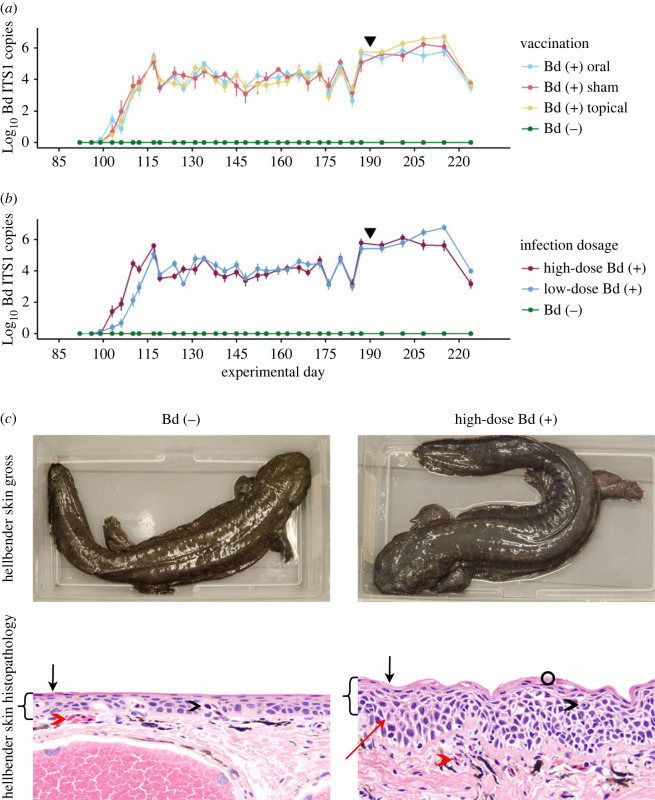
Pathogen load and host skin changes during experimental Bd infection of Eastern hellbender salamanders. (*a,b*) Bd load in experimentally infected hellbender salamanders at each longitudinal sampling event during experimental infection. Bd load is represented as mean and s.e. of Log_10_ Bd ITS1 copies in (*a*) all infected animals (merged low- and high-dose infection groups), summarized by vaccination group and (*b*) all vaccinated animals (merged sham, topical and oral vaccination groups), summarized by Bd infection dose. Animals were infected on experimental day 89. All Bd (+) hellbenders developed high pathogen burdens of greater than or equal to 1E5 Log_10_ Bd ITS1 copies within 28 days of infection and maintained high-Bd loads for the duration of the challenge. All control animals remained Bd (−) for the duration of the experiment. An incidental cold-shock occurred on experimental day 90, indicated by the black triangle. There was no overall effect of (*a*) vaccination or (*b*) Bd dose on infection intensity as measured by AUDPC. (*c*) Clinical chytridiomycosis in experimentally Bd-infected eastern hellbender salamanders. Skin coloration was altered by Bd infection; Bd (−) animals maintained normal brown skin coloration with clearly defined markings, Bd (+) animals developed diffuse opaque blue-grey skin coloration and exhibited increased skin sloughing. Hellbender epidermal structure was altered by Bd infection; the Bd (+) hellbender skin exhibits overall thickening of the epidermis (brackets), thickening of the exterior keratinized epidermis (black arrow) with abundant intracellular Bd (black circle), epidermal oedema (red arrow), and an increase in distance from dermal (red arrowhead) and epidermal (black arrowhead) capillaries to the surface of the skin. All histopathology images are magnified 200×.

All Bd-exposed animals acquired infection at the same rate, and the median time between infection and initial Bd detection was 21 days in all vaccination groups (figure 2*a*). Animals infected with a high dose of Bd tested Bd (+) with qPCR faster (median 17 days) than animals infected with a low dose of Bd (median 23 days). We identified no significant effect of vaccination on Bd load; there were no differences in infection intensity as represented by total Bd load during infection (median area AUDPC was 1.53 × 10^8^ for sham vaccinated, 2.56 × 10^8^ for topically vaccinated and 1.32 × 10^8^ for orally vaccinated; figure 2*a*; electronic supplementary material, table S3). There were no differences in infection intensity between high- and low-dose infection groups (median AUDPC = 1.71 × 10^8^ for high-dose-infected animals and 2.29 × 10^8^ for low-dose-infected animals, figure 2*a*; electronic supplementary material, table S3). All Bd (+) animals developed skin changes consistent with clinical chytridiomycosis including observed blue-grey colour change, and histologic evidence of epidermal hyperplasia and hyperkeratosis, regardless of vaccination treatment (figure 2*b*). There was no difference in survival by vaccination group; 97% (62/64) of Bd (+) animals survived with clinical disease for 135 DPI. The limited mortality included one animal that was found dead on experimental day 200 (111 DPI) with a high-Bd load of 6 × 10^7^ Bd ITS1 copies and one animal that was euthanized for humane reasons after receiving a severe wound from a conspecific on experimental day 173 (84 DPI). All Bd (+) hellbenders had skin changes characteristic of chytridiomycosis, as previously described, but no other significant lesions were noted on post-mortem examinations. No Bd (−) hellbenders tested positive for Bd ITS1 on qPCR or exhibited skin changes characteristic of clinical chytridiomycosis at any point in the experiment.

### 3.2. Host function in vaccination and infection

The hellbender skin metatranscriptome was characterized with over 4.53 billion high-quality mRNA reads from 70 samples which passed quality control and trimming processing steps (GenBank SRA Accession PRJNA700070; electronic supplementary material, table S4). RNA sequencing failed for two sham-vaccinated animals at the post-vaccination time point due to low quality of total RNA extracts; those samples were removed from further analysis. After filtering high-quality mRNA sequences from Bd and the microbiome, the hellbender skin transcriptome was assembled from 4.45 billion high-quality mRNA reads (average 60.2 million reads per sample). We filtered out low-expression transcripts (at least two reads per million mappable reads in at least two samples), resulting in a final dataset of 1 238 691 transcripts, each interpreted as equivalent to a gene or gene isoform. Of the assembled genes, 261 535 (21.11%) had a significant BLAST match, and of genes with a successful BLAST hit, 149 873 (57.31%) were successfully annotated with a GO term. We identified no clustering of global hellbender skin function among experimental groups or over time (electronic supplementary material, figure S1).

**Figure 3.**
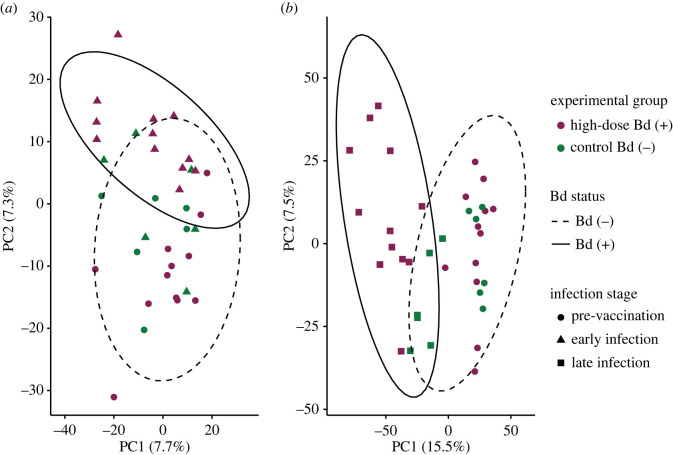
Bd infection alters hellbender skin gene expression at both (*a*) early and (*b*) late infection. Principal components analysis of DEGs identified in pairwise comparisons of high-dose Bd-infected (*n* = 12) and sham Bd-infected control (*n* = 6) hellbenders. Each plot represents gene expression comparisons between pre-vaccination (day 0) and either early infection (day 117, (*a*)) or late infection (day 187, (*b*)). Expression values are measured as Log_2_ transformed counts per million transcripts. Ellipses indicate 95% CI of expression values by Bd status: Bd (−) includes all animals at pre-vaccination and control animals at both early and late infection, Bd (+) includes high-dose Bd-infected animals at both early and late infection.

We compared the effect of vaccination and subsequent infection on the function of hellbender skin with DGE analysis. Using pairwise comparisons between vaccinated animals and unvaccinated control animals at the post-vaccination time point, we identified vaccine-specific changes to hellbender skin function in 641 DEGs (electronic supplementary material, table S1). We characterized changes in hellbender skin function in response to early and late infection by comparing gene expression in Bd (+) animals at pre-vaccination with both early and late infection respectively, identifying 1701 DEGs during Bd infection (figure 3; electronic supplementary material, table S1). Finally, we identified vaccine-specific effects of Bd infection on hellbender skin function with pairwise comparisons of Bd (+) and Bd (−) animals in each vaccination treatment during both early and late infection, revealing 2098 DEGs (electronic supplementary material, table S1).

**Figure 4.**
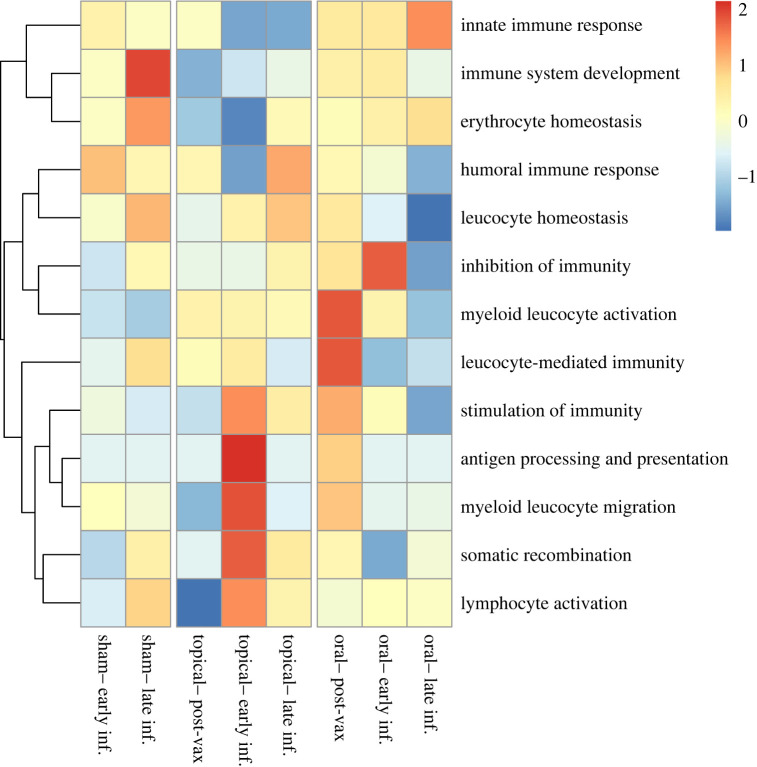
Hellbender skin immune response to vaccination and experimental Bd infection differs by vaccination strategy. Heatmap of differential immune gene expression in the skin of experimentally vaccinated and Bd-infected Eastern hellbender salamanders (FDR-corrected *p* < 0.05). DGE was characterized longitudinally by comparing expression of sham-vaccinated and sham Bd-infected (*n* = 6) hellbender skin with sham-vaccinated and high-dose Bd-infected (*n* = 6), topically vaccinated and high-dose Bd-infected (*n* = 3) and orally vaccinated and high-dose Bd-infected (*n* = 3) hellbender skin. Comparisons were performed at post-vaccination (day 89), early infection (day 117) and late infection (day 187). Expression is summarized by scaled and centred *z-*scores of Log_2_ average FC in all animals within an experimental group at each experimental time point. Immune gene function is parsed by child GO terms within Immune System Processes (GO:0002376). Sham-vaccinated animals do not show significant immune activation in early infection but show robust immune DGE in late infection. Topically vaccinated animals do not show significant immune DGE after vaccination. Topically vaccinated animals have robust DGE in antigen processing and presentation and leucocyte migration in early infection, and moderate immune DGE in late infection. Orally vaccinated animals show moderate immune DGE after vaccination and immune downregulation in early and late infection.

**Figure 5.**
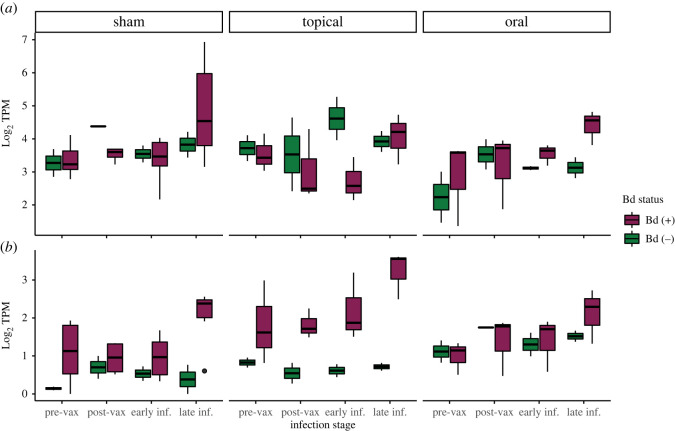
MHC gene expression in hellbender skin is affected by experimental vaccination and Bd infection. The expression of two differentially expressed MHC genes is represented as Log_2_ transcripts per million mapped reads (TPM, FDR-corrected *p* < 0.05). Expression is summarized at pre-vaccination (day 0), post-vaccination (day 89), early infection (day 117) and late infection (day 187) in each of six experimental groups: sham vaccinated and sham Bd infected (*n* = 2), sham vaccinated and high-dose Bd infected (*n* = 6), topical vaccinated and sham Bd infected (*n* = 2), topical vaccinated and high-dose Bd infected (*n* = 3), oral vaccinated and sham Bd infected (*n* = 2), and oral vaccinated and oral Bd infected (*n* = 3). The expression of MHC Class I gene isoform 18 (*a*) is affected by experimental time point and Bd status. The expression of MHC Class IIβ gene (*b*) is affected by experimental time point, Bd status, vaccination strategy and the interactions between time and Bd status, and Bd status and vaccination strategy.

To assess the specific effects of vaccination and infection on hellbender skin immunity, we tested for functional enrichment of immune-associated GO terms within DEGs. Genes with significant differential expression were functionally enriched for immune system processes relating to the development, activation and regulation of the immune system throughout the course of vaccination and infection (figure 4; electronic supplementary material, tables S5 and S6). To quantify enrichment, we filtered DEGs by predicted ontology and identified 104 immunological DEGs. Finally, to evaluate changes in MHC expression, a gene critical for Bd response in other species, we filtered immunological DEGs associated with the MHC and tested for effects of vaccination and infection on MHC expression (figure 5; electronic supplementary material, table S7). We found that two predicted isoforms of the MHC Class I gene DN14290 were differentially expressed in this experiment; isoform 18 expression was affected by both experimental day (ANOVA-type statistic (ATS) = 3.88, d.f. = 2.14, *p* = 0.0182) and the interaction between experimental day and Bd status (ATS = 3.07, d.f. = 2.14, *p* = 0.0427), and isoform 23 expression was affected by experimental day (ATS = 4.58, d.f. = 1.97, *p* = 0.0106). Expression of one differentially expressed MHC Class II*β* gene DN4350 was affected by vaccination (ATS = 4.90, d.f. = 1.89, *p* = 0.0086), Bd status (ATS = 11.6, d.f. = 1.00, *p* = 0.0006), experimental day (ATS = 5.42, d.f. = 2.27, *p* = 0.0030), and the interactions between vaccination and infection (ATS = 4.02, d.f. = 1.89, *p* = 0.0197), and between infection and experimental day (ATS = 3.38, d.f. = 2.27, *p* = 0.0283). However, despite observation of changes in immune regulation due to vaccination, especially oral vaccination, there was no difference among groups in disease progression as measured by Bd load or skin pathology.

### 3.3. Pathogen function in vaccination and infection

**Figure 6.**
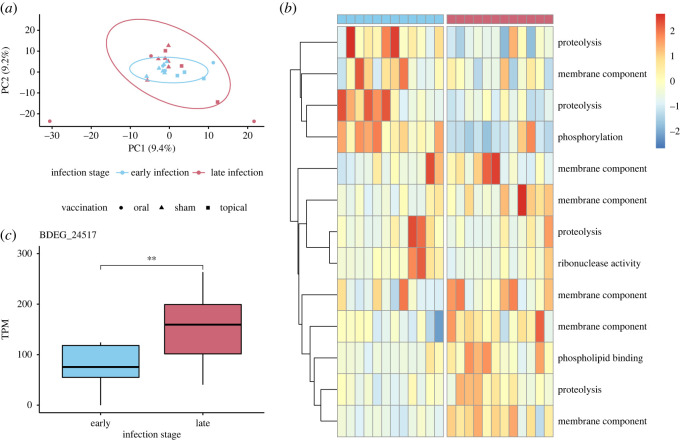
Bd gene expression varies by infection stage. (*a*) Principal components analysis of Bd gene expression in early and late infection summarized by Log_2_ transformed counts per million mapped reads. Ellipses indicate 95% CI for expression in early and late infection. (*b*) Heatmap of functionally annotated DEGs in early and late infection (FDR-corrected *p* < 0.05). Expression is summarized by scaled and centred *z*-scores of Log_2_ average TPM. Each row represents one DEG, and each column represents Bd function in a single animal in early or late infection. Columns headed in blue (left) represent Bd gene expression in early infection and columns headed in pink (right) represent Bd gene expression in late infection. (*c*) The expression of putative pathogenesis-associated gene BDEG_24517 is expressed significantly higher in late infection.

The Bd transcriptome was generated with 22.7 million high-quality Bd mRNA reads (average 947 637 reads per sample), which mapped to 8763 Bd genes in the JEL423 genome. We functionally annotated 7551 (75.42% of successfully mapped) genes; all were retained for further analysis. We used principal components analysis to characterize global Bd gene expression and found that Bd gene expression in early infection clustered within expression in late infection (figure 6*a*). Two outlier samples identified in the PCA represent orally vaccinated animals in late infection.

In order to assess the effect of infection stage on Bd gene expression, we compared expression in all Bd samples at late infection with expression at early infection and identified 51 DEGs (electronic supplementary material, table S2). We additionally evaluated the effect of vaccination on Bd gene expression by comparing expression in each vaccination group at late infection with expression at early infection. We identified a total of 75 DEGs between early and late infection (electronic supplementary material, table S2), of which 13 (17.33%) were functionally annotated. The expression of DEGs clustered by infection stage and not vaccination status (figure 6*b*). To identify functional changes in Bd expression between early and late infection, we tested for functional enrichment in DEGs. Proteolysis (GO:0006508) was the most frequently represented function of DEGs; 17% of annotated DEGs are associated with proteolysis. We also identified significant enrichment in late infection for a function involved in Bd pathogenesis: metallopeptidase activity (GO:0008237). At the gene level, we detected one DEG associated with Bd pathogenesis, a serine-type peptidase (BDEG_24517, figure 6*c*). The expression of BDEG_24517 was significantly upregulated in late infection (*t* = 3.11, d.f. = 11, *p* = 0.0099). There was no significant difference in the expression of BDEG_24517 between early and late infection among vaccination groups (*H* = 7.83, d.f. = 5, *p* = 0.17).

### 3.4. Host-associated microbiome characterization and function in vaccination and infection

#### 3.4.1. Host-associated microbiome community composition

We characterized the community composition of the hellbender skin microbiome with 4 613 037 high-quality reads originating from 140 samples (381–192 021 reads per sample). We identified 793 OTUs, which were interpreted as a proxy for bacterial species. The most frequently observed bacterial phyla in the hellbender microbiome were Proteobacteria (83%), Bacteroidetes (5.7%) and Firmicutes (1.2%). Microbiome alpha diversity differed by experimental time point with significant increases from pre-vaccination observed at post-vaccination (*V* = 1, *p* = 1.49 × 10^−8^), early infection (*V* = 8, *p* = 1.49 × 10^−7^) and late infection (*V* = 15, *p* = 1.02 × 10^−6^), but was not affected by vaccination, Bd infection, or the interaction between vaccination and infection (electronic supplementary material, table S8).

**Figure 7.**
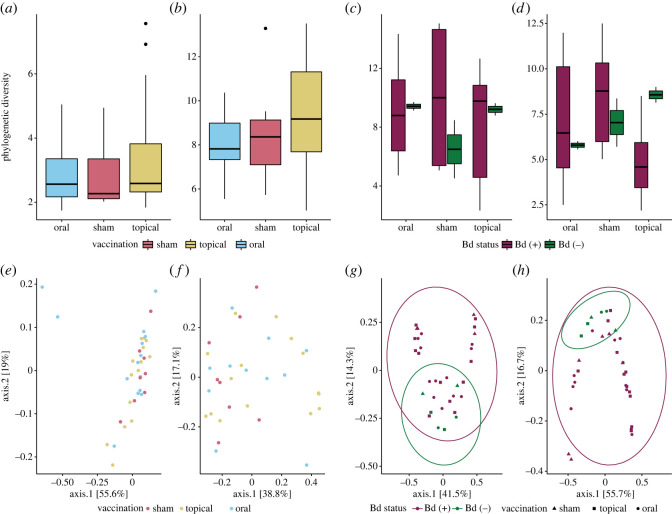
Community effects of vaccination and infection on host-associated microbiota. (*a*) Within-community diversity as measured by Faith's PD index is not significantly different between vaccination groups pre-vaccination (Kruskal–Wallis *χ*^2^ = 0.966, d.f. = 2, *p* = 0.617), or (*b*) post-vaccination (Kruskal–Wallis *χ*^2^ = 1.95, d.f. = 2, *p* = 0.378). (*c*) Early infection within-community diversity as measured by Faith's PD index is not significantly affected by vaccination treatment (Kruskal–Wallis *χ*^2^ = 0.112, d.f. = 2, *p* = 0.946) or Bd status (Kruskal–Wallis *χ*^2^ = 0.165, d.f. = 1, *p* = 0.684). (*d*) Late infection diversity was not significantly affected by vaccination (Kruskal–Wallis *χ*^2^ = 4.13, d.f. = 2, *p* = 0.127) or Bd status (Kruskal–Wallis *χ*^2^ = 0.521, d.f. = 1, *p* = 0.471). (*e*) Between-community diversity as measured with the Bray–Curtis distance is not significantly affected by vaccination groups pre-vaccination (PERMANOVA *p* = 0.421), or (*f*) in post-vaccination (PERMANOVA *p* = 0.231). (*g*) Early infection community composition as shown in the principal components analysis using Bray–Curtis distance is affected by Bd infection (PERMANOVA *p* = 0.032) but not by vaccination (PERMANOVA *p* = 0.814). Shown are 95% confidence ellipses for community structure in Bd (+) and Bd (−) hellbender microbiomes. (*h*) Late infection community composition was affected by Bd infection (PERMANOVA *p* = 0.029) but not vaccination (PERMANOVA *p* = 0.143).

To identify dynamic effects of vaccination and infection on the hellbender skin microbiome, we compared microbial community diversity indices and variance at each experimental time point. We did not observe any differences in Faith's PD (index or variance) or community composition among vaccination groups at pre-vaccination or post-vaccination (figure 7; electronic supplementary material, tables S9 and S10). Bd infection did not affect Faith's PD (index or variance) at either early or late infection (figure 7*c,d*; electronic supplementary material, table S9), but did affect skin microbiome community composition in early infection (*F* = 2.56, *R*^2^ = 0.0777, *p* = 0.03, figure 7*g*) and late infection (*F* = 3.21, *R*^2^ = 0.0815, *p* = 0.029, figure 7*h*; electronic supplementary material, table S10).

Finally, to evaluate the role of anti-Bd microbes on pathogen load, we tested for relationships between diversity and abundance of anti-Bd taxa. We found no correlation between Bd load and the diversity of anti-Bd bacterial OTUs in early infection (Spearman's rank correlation *ρ* = 0.0431, *S* = 3496, *p* = 0.828; electronic supplementary material, figure S2A) or late infection (Spearman's rank correlation *ρ* = 0.272, *S* = 3271, *p* = 0.146; electronic supplementary material, figure S2B). Similarly, abundance of anti-Bd bacterial taxa did not correlate to pathogen loads in early infection (Spearman's rank correlation *ρ* = −0.0060, *S* = 3676, *p* = 0.977) or late infection (Spearman's rank correlation *ρ* = −0.261, *S* = 5670, *p* = 0.163, data not shown).

#### 3.4.2. Host-associated microbiome function

**Figure 8.**
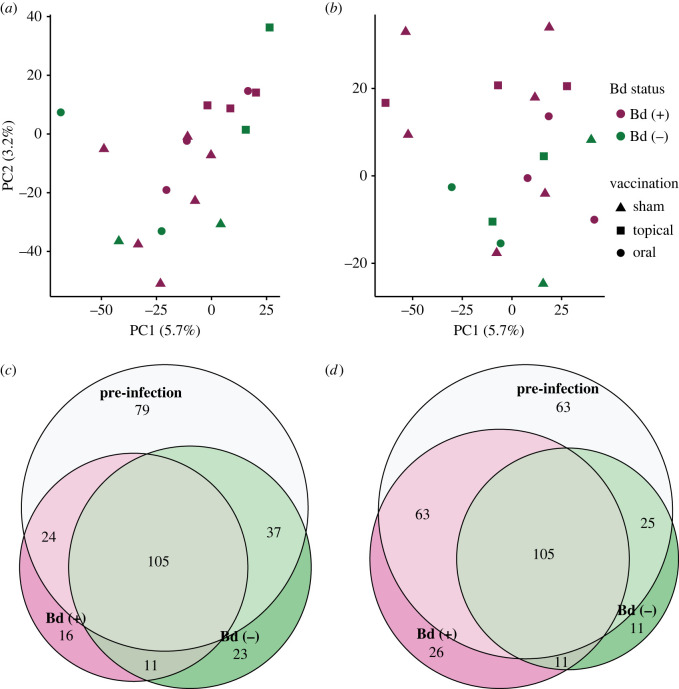
Hellbender skin microbiome function is not affected by Bd in early or late infection. (*a*) Principal components analysis of microbial gene expression in early infection and (*b*) late infection is summarized by Log_2_ transformed counts per million mapped reads. Expression is highly conserved in the commensal skin microbial community in early infection and late infection. (*c*) Microbiome function (as represented with GO terms) is compared between baseline pre-infection time points (both pre-vaccination and post-vaccination, pooled), early infection Bd (+) communities and early infection Bd (−) communities. (*d*) Microbiome function (as represented with GO terms) is compared between baseline pre-infection time points (both pre-vaccination and post-vaccination, pooled), late infection Bd (+) communities and late infection Bd (−) communities.

The hellbender commensal microbial metatranscriptome was assembled from 26.6 million high-quality mRNA reads (average 360 524 reads per sample) and consisted of 128 999 contigs, each roughly equivalent to a gene or gene isoform. Due to the relatively small per-sample prokaryote read counts, the lack of clustering observed in prokaryote gene expression (electronic supplementary material, figure S3), and the observation that hellbender commensal microbiome structure and diversity was affected by Bd infection but not vaccination, we focused our analysis on characterizing the unique functions of the microbiome in early and late infection of both Bd (+) and Bd (−) control hellbenders (figure 8; electronic supplementary material, table S11). Microbiome function was highly similar among experimental groups at all experimental time points and focused on metabolic processes, biological regulation and transport (electronic supplementary material, figure S3). Microbiome functions observed in Bd (+) communities during early and late infection were partially redundant; 13 functions were identified in both early and late infection (72% of early infection functions and 34% of late infection functions). Collectively, these findings indicate that the hellbender microbiome is altered by Bd infections, but though there was substantial variation in the diversity and abundance of anti-Bd skin bacteria found on hellbenders, there was no evidence that the microbiome itself was affecting disease progression.

## 4. Discussion

In this study, we evaluated the dynamic roles of host, pathogen and microbiome in vaccination and infection of a globally disseminated infectious disease. We found that vaccination with killed Bd did not protect against infection, reduce pathogen burden, prevent morbidity or affect survival in vaccinated animals compared with controls. Though hosts demonstrated diverse patterns of immune gene activation, these immune functions did not curtail infection. Pathogen function was dynamic; we saw increased expression of pathogenesis-associated genes in late infection. We found that the host-associated microbiome was affected by, but did not in turn affect infection status.

### 4.1. Host response to experimental vaccination

We identified no effect of topical or oral killed Bd vaccination on Bd infection intensity or survival in hellbender salamanders. However, at the post-vaccination time point, vaccinated hellbender skin function differed from both (i) the pre-vaccination time point and (ii) sham-vaccinated hellbenders at post-vaccination, in genes with predicted immune function. Orally vaccinated hellbenders downregulated genes with predicted immune function including inflammation and cytokine response, and robustly upregulated genes associated with protein transport, NK cell-mediated cytotoxicity, immunoglobulins and cytotoxic lymphocytes (figure 4; electronic supplementary material, tables S5 and S6). Topically vaccinated hellbenders downregulated genes associated with cellular response including interleukins, and MHC Class II antigen presentation, and slightly upregulated genes associated with inflammation and NK cell-mediated cytotoxicity (figure 4; electronic supplementary material, tables S5 and S6).

In addition to the DEGs reported here, it is likely that further genes were differentially expressed in hellbender skin during vaccination and infection which we were not able to detect due to practical constraints on sample size. Increasing sample size in RNA-Seq experiments increases recall (fraction of DEGs identified) but decreases precision (fraction of DEGs correctly identified as DE), and genes with higher FC are more likely to be recalled [115,116]. The sample sizes and methods used here are expected to recall greater than or equal to 80% of DEGs with FC greater than or equal to 1 [116]. Furthermore, the methods used here are robust at controlling FDR, even with relatively small sample sizes, suggesting that the DGE observed is correctly characterized [116]. Therefore, while it is likely that we were unable to identify all DEGs (especially those of less than 1 FC, or with vaccination and infection interactions), the sample sizes and methods employed here are appropriate for broad characterization of DGE in hellbender skin.

Crucially, despite stimulation of immune function genes, vaccination did not prevent infection or associated skin pathology, or limit Bd load. This finding indicates that oral administration of killed Bd may stimulate both an innate and acquired immune response to Bd in hellbender skin, but this immune response does not reduce infection burdens. In other studies, host immune response to Bd has been documented but was not consistently associated with the mitigation of disease [24,32,34]. In amphibians, oral administration of antigens is associated with improved upregulation of the mucosal immunoglobulin isotype, IgX, compared with systemic inoculation [117]. Here, oral administration of a priming dose of killed Bd resulted in upregulation of immunoglobulin-associated genes and elicited a more vigorous immune response in the skin than topical administration; this presents a novel administration option for future Bd-priming research and conservation actions.

### 4.2. Host response to experimental challenge

In this controlled environment Bd (+) animals survived 135 days with clinical disease as demonstrated by (i) high-Bd loads with peaks ranging from 8.8 × 10^4^ to 4.9 × 10^8^ ITS1 copies, (ii) characteristic changes in skin colour from brown to blue-grey [118], and (iii) characteristic skin changes including hyperplasia, hyperkeratosis and oedema [13]. We found no evidence of skin changes in Bd (−) animals in this study. In the wild, hellbenders often have low Bd loads and clinical disease including skin changes is rare [57–59,61]. Our study demonstrates that captive hellbenders can die from chytridiomycosis but are quite tolerant of the disease, surviving over a long period of time with clinical disease signs. Amphibian mortality from chytridiomycosis is generally attributed to loss or reduction of critical skin functions [17]. In our examination of hellbender skin function, there was no evidence of global changes in skin gene expression between Bd (+) and Bd (−) animals (electronic supplementary material, figure S1).

The experimental challenge did alter immune gene expression in Bd (+) hellbender skin; genes related to skin integrity, innate immunity and acquired immunity were upregulated in both early and late infection. Skin integrity genes including wound healing and apoptotic processes were upregulated in Bd (+) hellbender skin, following expression patterns that are common in species that can survive Bd infection [33,47,52]. Innate immune functions including complement-associated genes were also upregulated in all Bd (+) hellbender skin. The complement pathway is upregulated in many Bd-resistant species, but is not consistently activated in response to infection in susceptible species [33,38,40]. Hellbender skin also responded to the infection with upregulation of acquired immune genes including the MHC. Acquired immunity in general, and the MHC in particular, are critical components of a successful amphibian response to Bd [20,24,119]. In other salamander species susceptible to Bd, increased MHC expression is found in animals that successfully clear infection [35]. Collectively, these results suggest that hellbender skin immune function is similar to other species which can survive Bd infection.

Vaccination treatment did not alter the specific immune functions of Bd (+) hellbender skin; the mechanisms of hellbender skin immunity were conserved both in vaccinated and unvaccinated animals and among vaccination treatments. However, we found vaccine-specific differences in the timing of skin immune activation during both early and late infection relative to pre-vaccination baseline, but this variation in immune function was not associated with differences in Bd load. In early infection, topically vaccinated animals upregulated genes with acquired immune functions including antigen processing and presentation, myeloid leucocyte migration and somatic recombination. By contrast, both sham- and orally vaccinated animals maintained or downregulated immune gene activity. Despite these differences in skin immunity, vaccination groups did not have significantly different Bd loads, which indicates that hellbender skin immune function did not prevent or mitigate high-load Bd infections during early infection.

Hellbender immune function was strongly affected by vaccination strategy in late infection. Sham-vaccinated animals showed robust immune gene expression, particularly in immune system development, while topically vaccinated animals showed slight upregulation of immune genes and orally vaccinated animals downregulated immune function. These three distinct immune functional profiles are associated with Bd loads that were not significantly different. In some species, a sustained immune activation associated with poor infection outcomes suggests a dysregulated immune response that may contribute to mortality [32,34,37]. By contrast, a suppressed late infection immune response, like those observed in the orally vaccinated treatment, is associated with improved disease outcomes in other species [33,37]. The early activation in hellbender skin immune genes with no associated change in Bd load indicates that the observed acquired immune response to Bd was ineffective during this experiment.

### 4.3. Pathogen response to experimental challenge

We found that global Bd gene expression differed over the course of experimental infection but was unaffected by vaccination treatment. This suggests that Bd function differed over time rather than in response to priming of the immune system of the host. Activation of the DEGs identified over time in Bd represents a likely mechanism for the increase in Bd load observed during late infection. Among Bd genes differentially expressed between early and late infection, genes that regulate proteolysis were most commonly observed. Specifically, these genes were functionally enriched for metallopeptidase activity, a function associated with Bd virulence [120,121]. One putative pathogenesis-associated gene, a serine-type peptidase BDEG_24517 [122], was significantly upregulated in late infection, suggesting that it plays an important role in increasing pathogen load. The Bd strain used in this study, JEL422, is known to express lower levels of serine-type peptidases and higher levels of membrane transport genes compared with other strains of the hypervirulent Bd-GPL [50,120,122], but it should be noted that JEL422 expression changed in many similar genes in response to infection stage in our study.

### 4.4. Microbiome response to experimental vaccination and challenge

A final objective of this study was to characterize the relationship between hellbender skin microbiome and Bd disease outcomes. Amphibian skin microbiomes play an important role in Bd infection and can function as a symbiotic component of host innate immunity [26,123]. The hellbender skin microbial communities at all time points primarily comprised taxa in the phylum Proteobacteria, similar to previously reported captive hellbender microbiomes [124]. Although host immune activation is known to affect amphibian skin microbiomes [19,125,126], immune activation of orally vaccinated hellbenders post-vaccination did not alter the hellbender skin microbiome. Bd infection affected the beta but not alpha diversity of the hellbender skin microbiome. Some amphibian skin microbial communities are significantly affected by Bd status of the host and are more impacted by higher Bd loads, while other microbiome community structures are not altered by pathogen presence [127–130]. In many systems, the effect of Bd on the microbiome can be greater than the effect of the microbiome on Bd [25,131] and may be associated with proteolysis from enzymes secreted by the fungus leading to premature cell death and an increased frequency of skin sloughing [24]. Other studies have observed that increased diversity and abundance of anti-Bd bacteria are associated with lower pathogen burdens [132,133]. However, despite high variation in the richness and abundance of putative anti-Bd bacterial taxa, we found no relationship between anti-Bd taxa and Bd load, indicating that anti-Bd OTUs are not likely to be responsible for limiting Bd infections in hellbenders and allowing hellbenders to survive with Bd infections.

In addition to characterizing the hellbender skin microbiome over time during vaccination and infection with Bd, we evaluated the function of the microbial community at each experimental stage. Anti-Bd bacteria are known to alter gene expression in the presence of Bd; increased expression of nitrate reductase, putative oxalate-formate antiporter coding and acyl-coenzyme A dehydrogenase have been reported in anti-Bd bacteria grown with Bd [134]. We identified the expression of metabolic and regulatory pathways in the hellbender microbial metatranscriptome across all stages of the vaccination and challenge experiment, but did not detect the specific functions previously associated with anti-Bd microbial function in the presence of Bd. There were no alternative functions uniquely identified in the metatranscriptome after Bd infection to suggest new expression of metabolic or regulatory genes in response to Bd. However, we were not able to measure differential expression in core metabolic and regulatory processes between experimental days due to the relatively low read count of metatranscriptome genes identified in RNA sequencing. Although we can identify the expression of genes necessary for bacterial persistence in the presence of Bd, we could not definitively conclude that the hellbender microbiome responded functionally to Bd infection.

Bd can be inhibited by antifungal metabolites produced by the microbiome in other species [26,135,136]. This has led to great interest in the application of putative anti-Bd bacterial taxa or metabolites to susceptible species as a probiotic to protect hosts from Bd [29,123,137–140]. While it has been speculated that skin microbiomes may partially explain Bd tolerance in some salamander species [141], our results suggest that despite containing many putative anti-Bd skin microbes, the pre-existing skin microbiome is not a key factor accounting for control of Bd infection in hellbenders.

## 5. Conclusion and future directions

Generally, host immune function can mitigate the impacts of disease through two different mechanisms: resistance or tolerance. Both mechanisms have a potential ‘cost’ to the host which drives the evolutionary pressure for one or the other in a given host–pathogen system. Disease resistance is characterized by clearance of the pathogen from host tissues by the host immune system. Disease tolerance (distinct from immune tolerance to an innocuous antigen) permits pathogen presence so long as the health of the host is not significantly impacted [142,143]. We hypothesized that acquired immunity would allow hellbenders to resist Bd infections. As this experiment was time censored and we did not observe the final outcome of Bd infection, it was not possible to conclude whether hellbenders would have ultimately cleared the infection. However, despite mounting an acquired immune response to Bd, hellbenders were unsuccessful at limiting pathogen burden and experienced clinical disease regardless of the degree of immune gene activation. Prolonged immune responses themselves may add to disease impacts on the host, adding to the costs of disease resistance over tolerance. In other amphibian species, sustained immune activation contributed to susceptibility to Bd, and a relative reduction in immune activation against Bd prevented immune dysregulation and death [37]. The reduction in immune activation observed in early and late infection of orally vaccinated hellbenders may therefore represent a mechanism for disease tolerance in hellbenders.

Application of this longitudinal metatransciptome approach to study the relationship between host, pathogen and microbiome sheds important light on the mechanisms by which hellbenders survive Bd infection. This would not have been possible if these factors had been studied independently, or without repeated sampling throughout the course of infection. Without the examination of host response, we would not have been able to determine whether immune priming was successful or that the oral vaccination method was more effective than the topical vaccination. Immune priming ultimately did not affect pathogen load or disease outcome in this experiment, indicating that adult animals exposed to live Bd may not be able to clear the infection, even if vaccinated with killed Bd. This finding of sustained clinical disease is distinct from characteristic asymptomatic Bd infections in wild hellbenders. However, this clarifies that hellbenders can survive clinical disease under controlled conditions and may eventually downregulate immune functions that are associated with mortality in other amphibians [32,34,37]. Under conditions of stress, even species not normally considered susceptible to Bd may succumb to the disease [144]. A similar mechanism explains hellbenders' ability to tolerate disease and suggests that innate immune host factors other than the skin microbiome may be limiting pathogen growth.

## Data Availability

All sequencing data presented here are openly available in GenBank SRA Accession PRJNA700070. All other supporting data are openly available in Smithsonian FigShare: https://doi.org/10.25573/data.c.5665159 [145]. Supplementary material is available online [146].
